# miR-486 Promotes the Invasion and Cell Cycle Progression of Ovarian Cancer Cells by Targeting CADM1

**DOI:** 10.1155/2021/7407086

**Published:** 2021-08-05

**Authors:** Chenyang Li, Yue Wang, Hao Wang, Bowen Wang, Yunxia Wang, Nan Li, Yanli Qin, Yesheng Wang

**Affiliations:** ^1^Department of Gynecology, Henan Provincial People's Hospital, People's Hospital of Zhengzhou University, Zhengzhou, Henan 450003, China; ^2^The Third People's Hospital of Zhengzhou, Henan Province 450000, China; ^3^Henan University of Science and Technology, Henan Province 450000, China; ^4^Department of Hematology, Affiliated Cancer Hospital of Zhengzhou University, Henan Cancer Hospital, Zhengzhou 450008, China

## Abstract

**Objective:**

To explore the role and possible underlying mechanism of miR-486 in ovarian cancer (OC) cells.

**Methods:**

The expression of miR-486 and CADM1 was detected by qRT-PCR in OC tissues and adjacent nontumor tissues and OC cell lines. The dual-luciferase reporter gene system was used to determine the targeting relationship between miR-486 and CADM1. CCK-8, colony formation assay, Transwell, and flow cytometry were performed to detect cell proliferation, cell invasion, cell cycle progression, and the apoptotic cell death, respectively. Western blot was carried out to detect the expression of CADM1 protein and the proteins associated with cell cycle progression.

**Results:**

miR-486 was significantly upregulated in OC tissues and cells, while CADM1 expression was significantly downregulated. Dual-luciferase reporter assays further confirmed that CADM1 was a target gene of miR-486. Interference with miR-486 could inhibit the proliferation and invasion and promoted the apoptosis of SKOV3 cells. Knocking down both miR-486 and CADM1 significantly increased the SKOV3 cell proliferation, invasion, and the number of cells transitioning from the G0/G1 phase into the S phase of cell cycle and reduced the cellular apoptosis. Western blot analysis revealed that the expression of cell cycle progression-related proteins (CyclinD1, CyclinE, and CDK6) was significantly reduced, and the p21 expression was increased when interfering with both miR-486 and CADM1 expression.

**Conclusion:**

Our results suggested that miR-486 could act as a tumor promoter by targeting CADM1 and be a potential therapeutic target for the treatment of OC.

## 1. Introduction

Ovarian cancer (OC) ranks the third among the most common malignancies of the female reproductive system, with the highest mortality rate among malignant gynecological tumors [1]. Although the combined treatment of multiple disciplines such as surgery, chemoradiotherapy, and molecular target therapy is currently used in clinical practice, the incidence and mortality of OC remain high in China [2, 3]. OC has no significant early symptoms, no reliable tumor biological markers, and lack of effective therapeutic targets but is easy to recur, so its treatment has entered a bottleneck [4]. Local and systemic metastasis is the main cause of higher mortality in OC patients [5], but the specific mechanism of metastasis has not been clarified. A total of 92% histological type of OC is epithelial OC, of which high-grade serous ovarian cancer (HGSOC) accounts for more than two-thirds of reported cases [6]. Despite the different pathological types of tumors, surgical treatment combined with chemotherapy and other combined treatments are commonly applied, with no significant efficacy. OC still poses a serious threat to women's life and health. Therefore, in order to improve the prognosis of OC patients, it is essential to explore the potential mechanisms of tumor metastasis and identify new therapeutic targets.

MicroRNAs (miRNAs) are endogenous small noncoding RNAs with about 19 to 25 nucleotides in length. miRNAs can negatively regulate target gene mRNA expression and reduce the stability of mRNAs by binding to the 3′-untranslated region (3′-UTR) of target gene mRNA [7]. Despite their increasingly reported crucial roles in the mammalian genome [8], the dysregulation of miRNAs is closely related to the initiation and development of various types of tumors [9], including ovarian cancer [10]. For instance, miR-137 is downregulated in OC and forced expression of miR-137 can promote apoptosis of OC cells by inhibiting the expression of XIAP [11]; miR-125b slows OC progression by targeting SET protein to inhibit the epithelial-mesenchymal transition pathway [12]; miR-365 inhibits OC progression by targeting Wnt5a [13]; miR-18a inhibits OC growth by directly targeting TRIAP1 and IPMK [14]. Thus, miRNAs are closely related to the development of OC.

Recently, abnormal expression of miR-486 has been found in many related studies of tumors, which may be associated with tumor development. miR-486 is upregulated in OC, and some studies have found a close relationship between miR-486 and OC [15, 16]. Therefore, miR-486 may play an important role in the development and progression of OC. However, its detailed function and molecular mechanism in OC are still unclear and need to be further explored. Therefore, to determine the expression and biological function of miR-486 expression in OC, we first validated miR-486 expression in OC tissues. Subsequently, the effect of miR-486 expression on OC cell function and molecular mechanisms was examined by cellular assays. This study will provide a more comprehensive theoretical basis for miR-486 as a therapeutic target for OC.

## 2. Materials and Methods

### 2.1. Collection and Processing of Clinical Specimens

The tumor tissues and adjacent nontumor tissues were collected from the OC patients treated in Henan Provincial People's Hospital between January 2016 and December 2018. All patients had no chemotherapy or radiotherapy. For each patient, OC was the primary lesion, which was confirmed by pathological examination. None of the patients had any history of major systemic disease. Signed informed consent was obtained from each patient. This study was approved by the Medical Ethics Committee of Henan Provincial People's Hospital.

### 2.2. Cell Culture

The human normal ovarian epithelial cell line IOSE80 and human OC cell lines SKOV3, Caov3, ES-2, and OVCAR3 (American Type Culture Collection, USA) were used. All the cells were cultured in RPMI-1640 culture medium supplemented with 10% fetal bovine serum (FBS), 100 U/ml penicillin, and 100 *μ*g/ml streptomycin and incubated at 37°C with 5% CO_2_ and saturated humidity. The cells were digested with 0.25% trypsin every 2-3 days for passage, and the cells in the logarithmic phase were used for subsequent experiments.

### 2.3. Cell Transfection

miR-486 inhibitor (in-miR-486), Normal Control inhibitor (in-NC), siRNA NC (si-NC), and CADM1 siRNA (si-CADM1) were designed and synthesized by RiboBio (Guangzhou, China) and used in accordance with the manufacturer's instruction. SKOV3 cells were divided into five groups: in-miR-486 group, in-NC group, in-NC+si-NC group, in-miR-486+si-NC group, and in-miR-486+si-CADM1 group. SKOV3 cells were inoculated in 6-well plates at a density of 5 × 10^5^ cells/well to 50%-70% confluency. Then, they were transfected using Lipofectamine2000 (Invitrogen, USA) for 6 hours. After the transfection, the cells were rinsed three times with PBS, the culture medium was replaced with common culture medium, and then, culture continued for a further 48 hours. The transfection efficiency was verified using qRT-PCR, and the cells with successful transfection were used for subsequent experiments.

### 2.4. Cell Counting Kit-8 (CCK-8) Assay

The cells in the logarithmic growth phase were digested into single cell suspension and inoculated in 96-well plates at a density of 5 × 10^3^ cells/well. At 0, 24, 48, and 72 hours after transfection, the cell growth status was measured using CCK-8 (Beyotime Biotechnology, China). The plates were incubated for 1 hour added with 10 *μ*l of CCK-8 solution. Subsequently, the absorbance values were detected at 450 nm using a microplate reader (Bio-Rad Laboratory).

### 2.5. Colony Formation Assay

At 48 hours after transfection, SKOV3 cells in 6-well plates were digested with 0.25% trypsin and centrifuged at 1000 r/min for 5 min to resuspend and count. The cell suspension with gradient dilution was inoculated in the culture dish at appropriate cell density and the cells in even distribution at the bottom as possible. Then, they were cultured for 10 days until the colony is visible to the naked eye. The original culture medium was removed. The cells were rinsed with PBS once, added 10% formaldehyde for fixation for 15 min, rinsed with PBS twice, stained with crystal violet for 30 min, rinsed with PBS, dried, and finally photographed.

### 2.6. Transwell Assay

A total of 50 *μ*l serum-free medium was used to wet the polycarbonate membrane. The cells at 48 h after transfection were rinsed three times with serum-free medium. Then, 1 × 10^5^ cells/well were inoculated into the upper chamber with 100 *μ*l medium while 500 *μ*l complete medium was added to the lower chamber as chemokines for 12 h incubation. The culture was terminated when 5 cells passed to the lower chamber, and the polycarbonate membrane was removed. The invasive cells were rinsed with PBS three times, fixed in neutral formaldehyde for 10 min, and stained with hematoxylin. Their number was counted under a microscope in random five fields, and the mean value represented the cell invasion ability.

### 2.7. Flow Cytometry (FCM)

The transfected cells were digested and collected, and 5 × 10^4^-10 × 10^4^ cells were centrifuged at 1000 r/min for 5 min. The supernatant was aspirated, and then, 195 *μ*l of Annexin V-FITC binding solution was added to resuspend the cells. Subsequently, 5 *μ*l of Annexin V-FITC reagent was added, mixed gently, and placed at room temperature for 10 min in the dark. Another 10 *μ*l of propidium iodide (PI) staining solution was added and placed at room temperature for 10 min in the dark. Then, the cells were resuspended by adding 200 *μ*l of Annexin V-FITC binding solution. Apoptosis was detected using flow cytometry (FACSCanto TM II, BD Biosciences). In addition, 1 × 10^6^ cells were rinsed with PBS, fixed in precooled ethanol overnight at 4°C, and stained with PI after treatment with RNaseA (Sigma, USA). Finally, the cell cycle was analyzed by flow cytometry.

### 2.8. Dual-Luciferase Reporter Assay

Cell adhesion molecule 1 (CADM1) was predicted to be a target gene of miR-486 using Starbase V2.0, and their binding was verified by the luciferase reporter. The 3′-UTR of the wild-type (WT) CADM1 gene was cloned into the luciferase plasmid, and the mutant-type (MUT) plasmid was available from the mutation of CADM1 and miR-486 binding domain. SKOV3 cells were inoculated in 24-well plates to 60% confluency. The cells were transfected using Lipofectamine2000 according to the following groups: miR-486 mimics and WT plasmid containing CADM1 3′-UTR; NC mimics and WT plasmid containing CADM1 3′-UTR; miR-486 mimics and MUT plasmid containing CADM1 3′-UTR; and NC mimics and MUT plasmid containing CADM1 3′-UTR. After 48 hours of cotransfection, the relative luciferase activity levels of the different groups were measured using a dual-luciferase reporter assay system.

### 2.9. Quantitative Real-Time PCR (qRT-PCR)

Total RNA was isolated from OC tissues and cells using Trizol reagent (Invitrogen) according to the manufacturer's instruction. A total of 500 ng of RNA was reverse transcribed into cDNA using a cDNA transcription kit (ABI). The qRT-PCR reaction was performed using the SYBR Green PCR Master Mix (Invitrogen) with 40 cycles using the following parameters: 95°C for 2 min, 95°C for 15 s, and 60°C for 30 s. GAPDH or U6 was used as an internal reference. The relative expression levels were calculated using the 2^-*ΔΔ*Ct^ method. The primer sequences used were as follows: miR-486, forward: 5′-GAGTGTCGGGGCAGCTCAGT-3′ and reverse: 5′-GCAGGGTCCGAGGTATTC-3′; U6, forward: 5′-GCTTCGGCAGCACATATACTAAAAT-3′ and reverse: 5′-CGCTTCACGAATTTGCGTGTCAT-3′; CADM1, forward: 5′-GCAGGGTCCGAGGTATTC-3′ and reverse: 5′-CCACCAAGTCCCAAGATAGATA-3′; GAPDH, forward: 5′-AACGGATTTGGTCGTATTG-3′ and reverse: 5′-GGAAGATGGTGATGGGATT-3′.

### 2.10. Western Blot

At 48 hours after transfection, the cells were lysed on ice for 30 min using RIPA lysis buffer. The protein concentration was determined by a BCA protein assay kit (Beyotime Institute of Biotechnology, Inc., China). 40 *μ*g protein was separated using 10% SDS-PAGE with a voltage of 100 V and an electrophoresis time of 2 h, then transferred to a polyvinylidene difluoride (PVDF) membrane by wet transfer method, blocked with blocking solution at room temperature for 2 hours. After that, the membrane was incubated with rabbit polyclonal antibody CADM1 (1 : 1000, ab3910), rabbit monoclonal antibody p21 (1 : 1000, ab109520), rabbit monoclonal antibody CyclinD1 (1 : 1000, ab16663), rabbit monoclonal antibody CyclinE (1 : 1000, ab33911), rabbit monoclonal antibody cyclin-dependent kinase 6 (CDK6; 1 : 1000, ab124821), and rabbit polyclonal antibody GAPDH (1 : 1000, ab9485) overnight at 4°C. The next day, the membrane was rinsed with PBS for 30 min, incubated with horseradish peroxidase- (HRP-) labeled secondary antibody at 37°C for 1 hour, and rinsed with phosphate-buffered saline with Tween-20 (PBST) for 1 hour. Finally, the target protein was detected by enhanced chemiluminescence (ECL) kit (Beyotime, China), and the gray values were analyzed using ImageJ software.

### 2.11. Statistical Analysis

All experiments were repeated three times, and the experimental data were expressed as mean ± standard deviation (SD). SPSS22.0 software was used for statistical analysis. One-way analysis of variance (ANOVA) followed by Tukey's post hoc test was applied for the comparison of multiple groups, while *t*-test was applied for the comparison between two groups. *P* < 0.05 indicated a statistically significant test result.

## 3. Results

### 3.1. miR-486 Is Highly Expressed in OC Tissues and Cells

**Figure 1:**
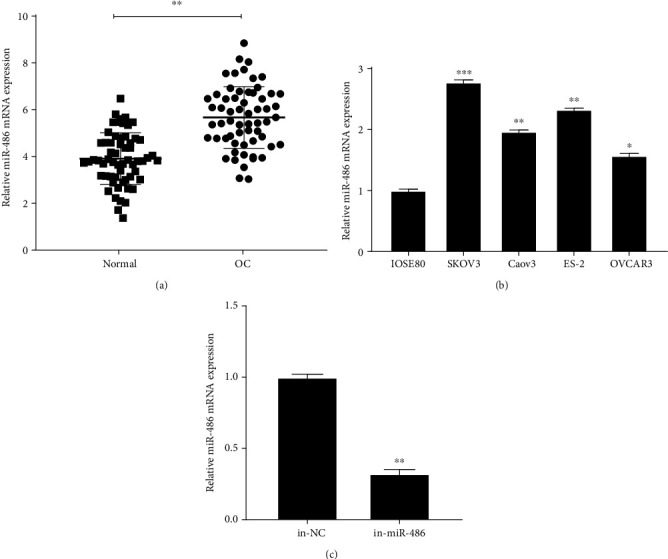
miR-486 is highly expressed in OC tissues and cells. (a) qRT-PCR to detect the expression of miR-486 in the OC tissues and the normal tissues. MiR-486 is highly expressed in OC tissues; (b) qRT-PCR to detect the expression of miR-486 in SKOV3, Caov3, ES-2, OVCAR3, and IOSE80; (c) qRT-PCR to detect the expression of miR-486 in SKOV3 after transfection with miR-486 inhibitor. ^∗^*P* < 0.05, ^∗∗^*P* < 0.01, and ^∗∗∗^*P* < 0.001.

To confirm the aberrant expression of miR-486 in the OC tissue, we assessed the relative expression in comparison with normal counterparts using qRT-PCR. The results confirmed that miR-486 expression was significantly increased in OC tissues compared with that in normal tissues (Figure 1(a)). In addition, miR-486 expression was also detected in SKOV3, Caov3, ES-2, OVCAR3, and IOSE80. However, as shown in Figure 1(b), miR-486 expression was significantly increased in SKOV3, Caov3, ES-2, and OVCAR3 compared with that in the normal IOSE80 cells. These results showed that miR-486 may be involved in the development of OC. Since SKOV3 cell line showed the highest endogenous expression of miR-486, they were selected for subsequent experiments.  Figure 1(c) shows the successful knockdown of miR-486 using miR-486 inhibitor.

### 3.2. Interfering with miR-486 Expression Inhibits the Biological Function of OC Cells

**Figure 2:**
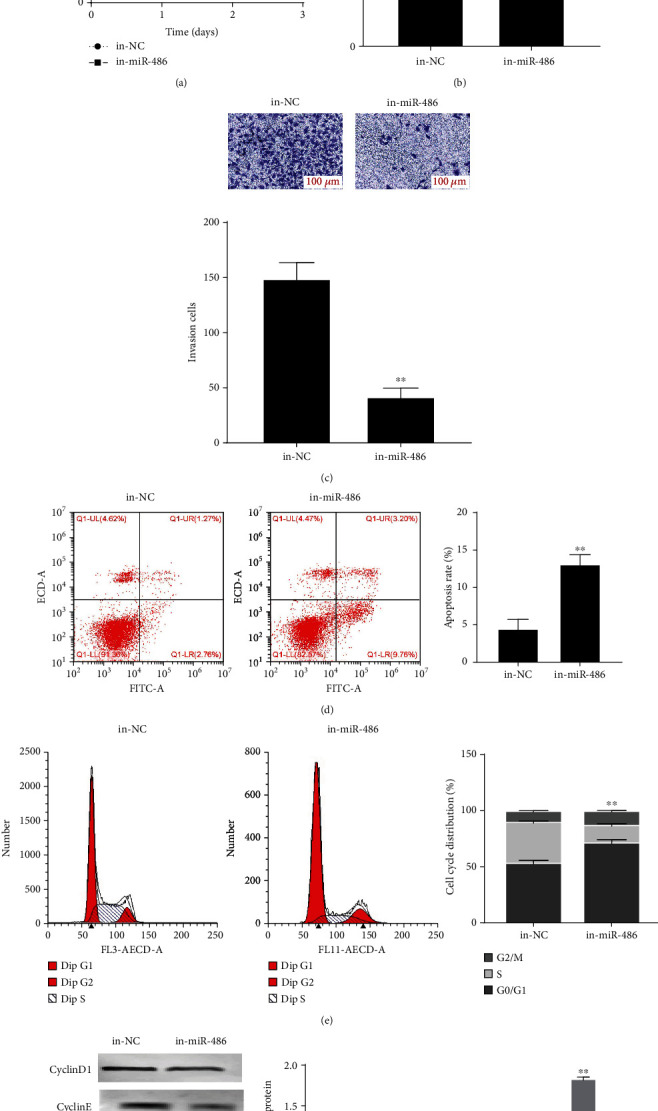
Interference with miR-486 expression inhibits the biological function of OC cells. (a) CCK-8 to detect the cell viability of SKOV3 after transfection with NC inhibitor and miR-486 inhibitor; (b) colony formation assay to detect the number of colonies of SKOV3 after transfection with NC inhibitor and miR-486 inhibitor; (c) Transwell assay to detect the invasion of SKOV3 after transfection with NC inhibitor and miR-486 inhibitor; (d, e) FCM to detect the apoptosis and cell cycle of SKOV3 after transfection with NC inhibitor and miR-486 inhibitor; (f)Western blot to detect the expression levels of cell cycle-related proteins p21, CyclinD1, CyclinE, and CDK6. ^∗^*P* < 0.05 and ^∗∗^*P* < 0.01 vs. the in-NC group.

To investigate the biological function of miR-486 in OC cells, after exogenously manipulating the miR-486 expression, CCK-8, colony formation assay, Transwell assay, and FCM were used to detect cell proliferation, invasion, and apoptosis, respectively. The results showed that in comparison with the in-NC group, cell viability and proliferation (Figures 2(a) and 2(b)) and invasion (Figure 2(c)) in the in-miR-486 group were significantly reduced. Flow cytometry analysis showed a significant increase in the apoptotic death in-miR486 group (Figure 2(d)) and an increased proportion of cells in the G0/G1 phase of the cell cycle with a concurrent reduction of cells in the S phase (Figure 2(e)). Western blot was used to detect the expression levels of proteins associated with cycle progression. The result showed that the protein expression of CyclinD1, CyclinE, and CDK6 was significantly decreased and p21 was significantly increased in the in-miR-486 group compared with the in-NC group (Figure 2(f)). The above results suggest that interference with miR-486 expression inhibited the biological function of OC cells.

### 3.3. CADM1 Is a Target of miR-486 in OC

**Figure 3:**
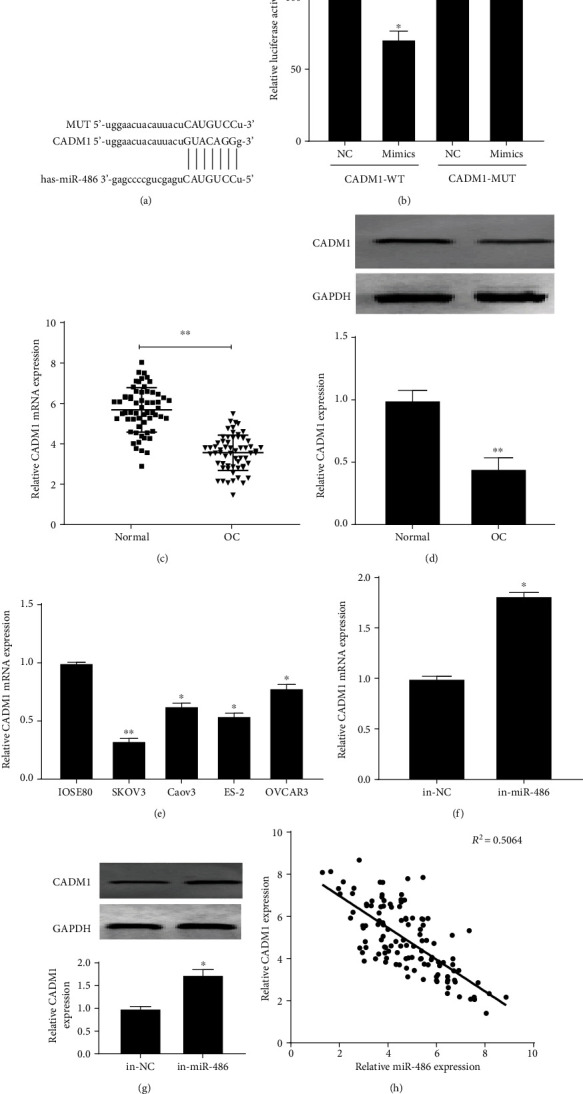
miR-486 targets CADM1. (a) Starbase V2.0 to predict the binding site between miR-486 and CAMD1; (b) dual-luciferase reporter assay to detect the targeting relationship between miR-486 and CADM1; (c) qRT-PCR assay to detect CADM1 expression in normal and OC tissues. CADM1 expression is low in OC tissues; (d) Western blot to detect the expression of CADM1 protein in normal and OC tissues; (e) qRT-PCR to detect the expression of CADM1 in SKOV3, Caov3, ES-2, OVCAR3, and IOSE80; (f) qRT-PCR to detect the expression of CADM1 mRNA in SKOV3 cells after transfection with miR-486 inhibitor; (g) Western blot to detect the expression of CADM1 protein in SKOV3 cells after transfection with miR-486 inhibitor; (h) Pearson analysis of the correlation between miR-486 and CADM1. ^∗^*P* < 0.05 and ^∗∗^*P* < 0.01.

miRNAs have been widely reported to repress the expression of target genes by binding to 3′-UTR [7]. Hence, we tried to find the targets regulated by miR-486. According to the prediction by Starbase V2.0 database, CADM1 could be one of the target genes of miR-486 (Figure 3(a)). To verify the targeting relationship between miR-486 and CADM1, WT or MUT containing CADM1 3′-UTR and miR-486 mimics or mimic NC were cotransfected in SKOV3 cells to detect luciferase activity. As shown in Figure 3(b), luciferase activity was significantly inhibited after cotransfection with miR-486 mimics and the WT CADM1 3′-UTR. The results of qRT-PCR and Western blot showed that mRNA and protein expression levels of CADM1 were significantly decreased in OC tissues and cells (Figures 3(c)–3(e)). Subsequently, we found that knocking down miR-486 significantly upregulated the mRNA and protein expression of CADM1 in SKOV3 cells (Figures 3(f) and 3(g)). The correlation analysis revealed that there was a negative correlation between miR-486 and CADM1 expression (Figure 3(h)). These results suggested that miR-486 regulated the expression level of CADM1 in OC.

### 3.4. miR-486 Regulates OC Proliferation, Viability, Survival, and Invasion through CADM1

**Figure 4:**
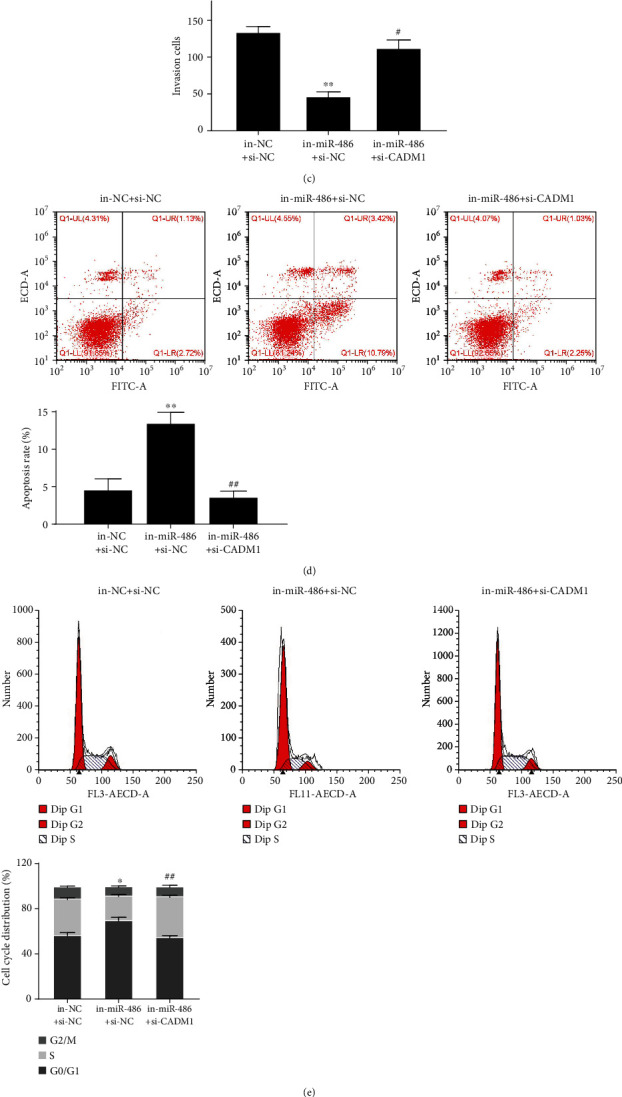
miR-486 downregulates CADM1 to be involved in the biological function of OC cells. (a) CCK-8 assay to detect the cell viability of SKOV3 after transfection with in-NC+si-NC, in-miR-486+si-NC, and in-miR-486+si-CADM1; (b) colony formation assay to detect the cell colony formation ability of SKOV3 after transfection with in-NC+si-NC, in-miR-486+si-NC, and in-miR-486+si-CADM1; (c) Transwell assay to detect the invasion ability of SKOV3 after transfection with in-NC + si-NC, in-miR-486+si-NC, and in-miR-486+si-CADM1; (d, e) FCM to detect the apoptosis rate and cell cycle of SKOV3 after transfection with in-NC+si-NC, in-miR-486+si-NC, and in-miR-486+si-CADM1; (f) Western blot to detect the expression levels of cell cycle-related proteins p21, CyclinD1, CyclinE, and CDK6. ^∗^*P* < 0.05 and ^∗∗^*P* < 0.01 vs. the in-NC+si-NC group; ^#^*P* < 0.05 and ^##^*P* < 0.01 vs. the in-miR-486+si-NC group.

To investigate whether miR-486 exerts its biological function in OC by regulating CADM1, the rescue experiment was carried out by transfecting in-miR-486, in-NC, si-NC, si-CADM1, and in-miR-486+si-CADM1 in SKOV3 cells. The results showed that cell proliferation, viability, and invasion were significantly reduced, apoptosis was significantly increased, and the proportion of cells in the G0/G1 phase was significantly increased, while in the S phase, it was significantly reduced in the in-miR-486 group (*P* < 0.05). However, knocking down CADM1 in the same group reversed the miR-486 inhibition effects on different OC hallmarks (Figures 4(a)–4(e)). In addition, as shown in Figure 4(f), compared with the in-NC+si-NC group, the protein expression levels of CyclinD1, CyclinE, and CDK6 were significantly decreased while the protein expression levels of CADM1 and p21 were significantly increased in the in-miR-486+si-NC group; compared with the in-miR-486+si-NC group, the protein expression levels of CyclinD1, CyclinE, and CDK6 were significantly increased while the protein expression levels of CADM1 and p21 were significantly decreased in the in-miR-486+si-CADM1 group (*P* < 0.05). These results indicated that miR-486 downregulates CADM1 expression to affect the biological function of SKOV3 cells.

## 4. Discussion

OC is a gynecological malignant tumor with the highest mortality rate among the three malignant tumors of the female reproductive system [1, 17]. With medical advances, the prognosis of OC patients has improved, but the 5-year survival rate is still low due to recurrence, drug resistance, early metastasis, and lack of effective targeted therapy [18]. Therefore, exploring the mechanism of OC hallmarks is essential for the new treatment strategies. In recent years, miRNAs have attracted attention of many scholars because of its importance in the early diagnosis, occurrence and development of tumors, and tumor cell invasion and migration [19, 20].

Previous studies have found that miR-486 is important in the development and prognosis of cancers [21]. miR-486 is expressed at low levels in a variety of malignant tumors, including oral tongue squamous cell carcinoma [22], cervical cancer [23], lung cancer [24], papillary thyroid carcinoma [25], and esophageal cancer [26]. However, it is also highly expressed in some tumors, such as colon cancer [27], hepatocellular carcinoma [28], and OC [15]. In this study, we found that miR-486 was upregulated in OC tissues and cells; at the same time, interfering with miR-486 inhibited the proliferation, invasion, and cell cycle of OC cells and promoted their apoptosis. These results suggest that miR-486 plays a biological role in promoting the development in OC.

Further, the underlying regulatory mechanism of miR-468 was also investigated. Ma et al. have found that estrogen receptor signaling downregulates miR-486-5p and upregulates the target gene OLFM4, thereby slowing the development and progression of OC [15]. In the present study, CADM1 was confirmed to be a direct target of miR-486. CADM1 is a member of immunoglobulin superfamily, which can prevent malignant transformation and metastasis through the maintenance of epithelial cells [29]. CADM1 gene on chromosome fragment 11q23.2 was originally identified as a tumor suppressor gene in non-small-cell lung cancer, which was confirmed in nude mice [30]. CADM1 encodes an immunoglobulin superfamily cell adhesion molecule expressed in the brain, testis, lung, and various epithelial tissues [31]. CADM1 protein plays an important role in epithelial cell adhesion through the homogenous transmission among adjacent cells [32]. However, its molecular mechanism in OC remains unclear. The results of cell rescue experiment confirmed that interference with CADM1 reversed the effect of miR-486 inhibition on OC cell functions. Therefore, it can be used as a biomarker and a potential therapeutic target in oncology. It has been reported that overexpression of CADM1 inhibits the progression of tumors [33, 34]. These results are consistent with our findings, indicating that miR-486 regulates the development and progression of OC by targeting CADM1.

At present, more and more scholars consider tumor as a kind of cellular periodic disease, which is closely related to a series of pathophysiological processes such as cell growth and proliferation, differentiation, apoptosis, repair, and carcinogenesis [35]. Cell cycle is a process tightly regulated by genes [36]; the abnormal cell cycle regulation will cause cell growth and proliferation out of control, resulting in tumor development or even metastasis [37, 38]. Cyclins, CDKs, and CDK inhibitors constitute a network system to regulate at the critical restriction points [39]. Among them, CyclinD1, CyclinE, and CDK6 are the key to promote the cell cycle from the G1 phase to the S phase and to initiate the operation of cell cycle, which are most closely related to tumors. They are currently recognized as oncogenes. Their abnormal expression and function lead to the disorder of cell cycle regulation and cell proliferation out of control and then cause tumor development and metastasis and even related to the treatment and prognosis of cancer patients [40–43], while the function of p21 is the opposite [44]. Our study found that interference with miR-486 can inhibit the expression of CyclinD1, CyclinE, and CDK6 while promoting the expression of p21 in OC cells. At the same time, reduction of CADM1 expression can reverse the corresponding expression of proteins.

In summary, miR-486 is highly expressed in OC cells and tissues and miR-486 targets CADM1 to regulate OC cell biological functions such as proliferation, invasion, and cycle progression. These conclusions also need to be verified by subsequent animal experiments, and the collection of clinical tissue to analyze the relationship between miR-486 expression and clinicopathology. This study suggests that miR-486 may be a potential target for the treatment of OC and has a good clinical application prospect for the prognosis of OC patients.

## Data Availability

The datasets supporting the conclusions of this article are included within the article.

## References

[1] Siegel R. L., Miller K. D., Goding Sauer A. (2020). Colorectal cancer statistics, 2020. *CA: a Cancer Journal for Clinicians*.

[2] Torre L. A., Trabert B., DeSantis C. E. (2018). Ovarian cancer statistics, 2018. *CA: a Cancer Journal for Clinicians*.

[3] Wang J., Li J., Chen R., Lu X. (2018). Contribution of lymph node staging method and prognostic factors in malignant ovarian sex cord-stromal tumors: a world wide database analysis. *European Journal of Surgical Oncology*.

[4] Zhao R., Liu Q., Lou C. (2018). MicroRNA-299-3p regulates proliferation, migration and invasion of human ovarian cancer cells by modulating the expression of OCT4. *Archives of Biochemistry and Biophysics*.

[5] Liu D. T., Yao H. R., Li Y. Y., Song Y. Y., Su M. Y. (2018). MicroRNA-19b promotes the migration and invasion of ovarian cancer cells by inhibiting the PTEN/AKT signaling pathway. *Oncology Letters*.

[6] Levanon K., Crum C., Drapkin R. (2008). New insights into the pathogenesis of serous ovarian cancer and its clinical impact. *Journal of Clinical Oncology*.

[7] Hausser J., Zavolan M. (2014). Identification and consequences of miRNA-target interactions -- beyond repression of gene expression. *Nature Reviews. Genetics*.

[8] Frith J. E., Kusuma G. D., Carthew J. (2018). Mechanically-sensitive miRNAs bias human mesenchymal stem cell fate via mTOR signalling. *Nature Communications*.

[9] Wang X. Z., Hang Y. K., Liu J. B., Hou Y. Q., Wang N., Wang M. J. (2016). Over-expression of microRNA-375 inhibits papillary thyroid carcinoma cell proliferation and induces cell apoptosis by targeting ERBB2. *Journal of Pharmacological Sciences*.

[10] Lei R., Xue M., Zhang L., Lin Z. (2017). Long noncoding RNA MALAT1-regulated microRNA 506 modulates ovarian cancer growth by targeting iASPP. *Oncotargets and Therapy*.

[11] Li X., Chen W., Zeng W., Wan C., Duan S., Jiang S. (2017). MicroRNA-137 promotes apoptosis in ovarian cancer cells via the regulation of XIAP. *British Journal of Cancer*.

[12] Ying X., Wei K., Lin Z. (2016). MicroRNA-125b suppresses ovarian cancer progression via suppression of the epithelial-mesenchymal transition pathway by targeting the SET protein. *Cellular Physiology and Biochemistry*.

[13] Wang Y., Xu C., Wang Y., Zhang X. (2017). MicroRNA-365 inhibits ovarian cancer progression by targeting Wnt5a. *American Journal of Cancer Research*.

[14] Liu P., Qi X., Bian C. (2017). MicroRNA-18a inhibits ovarian cancer growth via directly targeting TRIAP1 and IPMK. *Oncology Letters*.

[15] Ma H., Tian T., Liang S. (2016). Estrogen receptor-mediated miR-486-5p regulation of OLFM4 expression in ovarian cancer. *Oncotarget*.

[16] Nakamura N., Terai Y., Nunode M. (2020). The differential expression of miRNAs between ovarian endometrioma and endometriosis-associated ovarian cancer. *Journal of Ovarian Research*.

[17] Liu J., Jiang Y., Wan Y., Zhou S., Thapa S., Cheng W. (2018). MicroRNA-665 suppresses the growth and migration of ovarian cancer cells by targeting HOXA10. *Molecular Medicine Reports*.

[18] Cliby W. A., Powell M. A., al-Hammadi N. (2015). Ovarian cancer in the United States: contemporary patterns of care associated with improved survival. *Gynecologic Oncology*.

[19] Qu X., Gao D., Ren Q., Jiang X., Bai J., Sheng L. (2018). miR-211 inhibits proliferation, invasion and migration of cervical cancer via targeting SPARC. *Oncology Letters*.

[20] Jin B., Jin D., Zhuo Z., Zhang B., Chen K. (2020). MiR-1224-5p activates autophagy, cell invasion and inhibits epithelial-to-mesenchymal transition in osteosarcoma cells by directly targeting PLK1 through PI3K/AKT/mTOR signaling pathway. *OncoTargets and Therapy*.

[21] Jiang M., Li X., Quan X. (2018). MiR-486 as an effective biomarker in cancer diagnosis and prognosis: a systematic review and meta-analysis. *Oncotarget*.

[22] Chen Z., Yu T., Cabay R. J. (2017). miR-486-3p, miR-139-5p, and miR-21 as biomarkers for the detection of oral tongue squamous cell carcinoma. *Biomarkers in Cancer*.

[23] Ye H., Yu X., Xia J., Tang X., Tang L., Chen F. (2016). MiR-486-3p targeting ECM1 represses cell proliferation and metastasis in cervical cancer. *Biomedicine & Pharmacotherapy*.

[24] Fotinos A., Nagarajan N., Martins A. S. (2014). Bone morphogenetic protein-focused strategies to induce cytotoxicity in lung cancer cells. *Anticancer Research*.

[25] Swierniak M., Wojcicka A., Czetwertynska M. (2013). In-depth characterization of the microRNA transcriptome in normal thyroid and papillary thyroid carcinoma. *The Journal of Clinical Endocrinology and Metabolism*.

[26] Hummel R., Wang T., Watson D. I. (2011). Chemotherapy-induced modification of microRNA expression in esophageal cancer. *Oncology Reports*.

[27] Mosakhani N., Sarhadi V. K., Borze I. (2012). MicroRNA profiling differentiates colorectal cancer according to KRAS status. *Genes, Chromosomes & Cancer*.

[28] Huang Y. H., Lin K. H., Chen H. C. (2012). Identification of postoperative prognostic microRNA predictors in hepatocellular carcinoma. *PLoS One*.

[29] de Haan H. G., Bezemer I. D., Vossen C. Y. (2014). Genetic variants in Cell Adhesion Molecule 1 (CADM1): a validation study of a novel endothelial cell venous thrombosis risk factor. *Thrombosis Research*.

[30] Ishimura M., Sakurai-Yageta M., Maruyama T. (2012). Involvement of miR-214 and miR-375 in malignant features of non-small-cell lung cancer by down-regulating CADM1. *Journal of Cancer Therapy*.

[31] Kikuchi S., Iwai M., Sakurai-Yageta M. (2012). Expression of a splicing variant of the CADM1 specific to small cell lung cancer. *Cancer Science*.

[32] Sakurai-Yageta M., Masuda M., Tsuboi Y., Ito A., Murakami Y. (2009). Tumor suppressor CADM1 is involved in epithelial cell structure. *Biochemical and Biophysical Research Communications*.

[33] Saito M., Goto A., Abe N. (2018). Decreased expression of CADM1 and CADM4 are associated with advanced stage breast cancer. *Oncology Letters*.

[34] Yao J., Chen Y., Wang Y. (2014). Decreased expression of a novel lncRNA CADM1-AS1 is associated with poor prognosis in patients with clear cell renal cell carcinomas. *International Journal of Clinical and Experimental Pathology*.

[35] Mladenov E., Magin S., Soni A., Iliakis G. (2016). DNA double-strand-break repair in higher eukaryotes and its role in genomic instability and cancer: cell cycle and proliferation-dependent regulation. *Seminars in Cancer Biology*.

[36] Ma X., Huang M., Wang Z., Liu B., Zhu Z., Li C. (2016). ZHX1 inhibits gastric cancer cell growth through inducing cell-cycle arrest and apoptosis. *Journal of Cancer*.

[37] Prasedya E. S., Miyake M., Kobayashi D., Hazama A. (2016). Carrageenan delays cell cycle progression in human cancer cells in vitro demonstrated by FUCCI imaging. *BMC Complementary and Alternative Medicine*.

[38] Lehmann B. D., Bauer J. A., Chen X. (2011). Identification of human triple-negative breast cancer subtypes and preclinical models for selection of targeted therapies. *The Journal of Clinical Investigation*.

[39] Malumbres M., Barbacid M. (2009). Cell cycle, CDKs and cancer: a changing paradigm. *Nature Reviews. Cancer*.

[40] Xia B., Yang S., Liu T., Lou G. (2015). miR-211 suppresses epithelial ovarian cancer proliferation and cell-cycle progression by targeting Cyclin D1 and CDK6. *Molecular Cancer*.

[41] Li N., Zhong X., Lin X. (2012). Lin-28 homologue A (LIN28A) promotes cell cycle progression via regulation of cyclin-dependent kinase 2 (CDK2), cyclin D1 (CCND1), and cell division cycle 25 homolog A (CDC25A) expression in cancer. *The Journal of Biological Chemistry*.

[42] Deng M., Zeng C., Lu X. (2017). miR-218 suppresses gastric cancer cell cycle progression through the CDK6/Cyclin D1/E2F1 axis in a feedback loop. *Cancer Letters*.

[43] Shin S. S., Park S. S., Hwang B. (2016). MicroRNA-892b influences proliferation, migration and invasion of bladder cancer cells by mediating the p19ARF/cyclin D1/CDK6 and Sp-1/MMP-9 pathways. *Oncology Reports*.

[44] El-Deiry W. S. (2016). p21(WAF1) mediates cell-cycle inhibition, relevant to cancer suppression and therapy. *Cancer Research*.

