# Corrigendum to “miR-486 Promotes the Invasion and Cell Cycle Progression of Ovarian Cancer Cells by Targeting CADM1”

**DOI:** 10.1155/2021/9868462

**Published:** 2021-12-24

**Authors:** Hao Wang, Chenyang Li, Yunxia Wang, Nan Li, Yanli Qin, Yesheng Wang

**Affiliations:** ^1^The Third People's Hospital of Zhengzhou, Henan Province 450000, China; ^2^Department of Gynecology, Henan Provincial People's Hospital, People's Hospital of Zhengzhou University, Zhengzhou, Henan 450003, China; ^3^Department of Hematology, Affiliated Cancer Hospital of Zhengzhou University, Henan Cancer Hospital, Zhengzhou 450008, China

In the article titled “miR-486 Promotes the Invasion and Cell Cycle Progression of Ovarian Cancer Cells by Targeting CADM1” [1], the author list is revised as above by removing Yue Wang and Bowen Wang and updating the order of authors. This change is made with the agreement of all authors.
